# A question of persistence: Langerhans cells and graft-versus-host disease

**DOI:** 10.1111/exd.12325

**Published:** 2014-03-28

**Authors:** Matthew Collin, Laura Jardine

**Affiliations:** Human Dendritic Cell Laboratory, Institute of Cellular Medicine, Newcastle UniversityNewcastle upon Tyne, UK

**Keywords:** Graft-versus-host disease, Langerhans cells

## Abstract

Langerhans cells (LCs) have been scrutinized many times in studies of the pathogenesis of graft-versus-host disease (GVHD). As migratory dendritic cells, LCs are capable of direct antigen presentation to cytotoxic T cells. Their self-renewal capacity has led to speculation that persistent recipient LCs could provide a continuous source of host antigen to donor T cells infused during hematopoietic stem cell transplantation (HSCT). In this issue of Experimental Dermatology, a new study examines at the relationship between recipient LCs and chronic GVHD.

The role of Langerhans cells (LCs) in the pathogenesis of graft-versus-host disease (GVHD) remains enigmatic. LCs are the paradigmatic migratory dendritic cell (DC) and are potent at priming CD8+ T cells (1). In haematopoietic stem cell transplantation (HSCT), the potential of recipient LCs to promote GVHD by presenting host antigen to donor cytotoxic T cells was first proposed more than 30 years ago by Claude Perreault (2). More recent enquiry has been galvanized by two important observations: first, that deletion of recipient antigen-presenting cell function can ablate GVHD in mice (3), and second, that LCs proliferate locally and maintain homeostasis independently of the bone marrow, even after myeloablative transplantation (4,5).

Human studies had previously shown that LCs were in cell cycle (6) and understanding the turnover of LCs, especially after reduced intensity transplantation, became a pivotal question. Several studies asked whether persistent recipient LCs were associated with an increased risk of acute GVHD and might therefore offer a new target of therapeutic intervention (7,8). However, a key factor was overlooked, namely that acute GVHD itself may cause sufficient cutaneous inflammation to deliver a knockout blow to resident LCs, resulting in the recruitment of donor-derived cells (5). This presented a paradox: the very ‘risk factor’ for GVHD, a high proportion of persistent recipient LCs, is more likely to be observed in the absence of GVHD. Another way to consider this is that recipient LCs, although self-renewing, are actually self-limiting in terms of priming donor T cells: the more inflammation that results, the more likely they are to disappear (Fig. 1).

**Figure 1:**
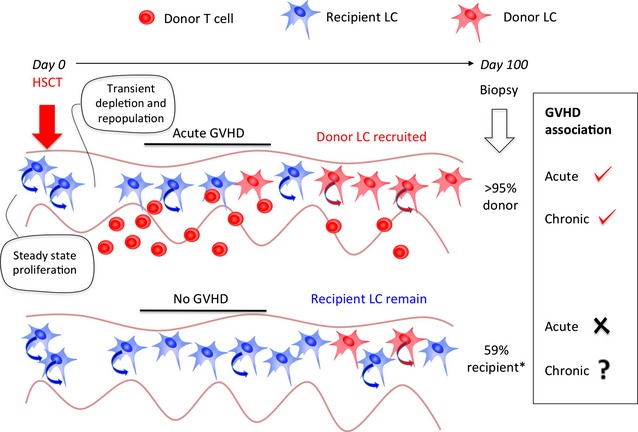
Association of GVHD with donor LC engraftment. LCs are self-renewing in the steady state. Conditioning with chemo/radiotherapy for HSCT leads to transient depletion and repopulation, probably by local proliferation. Inflammation caused by acute GVHD leads to loss of recipient LCs and engraftment of donor cells (also self-renewing). This means that a biopsy taken after acute GVHD is likely to find an inverse association between the persistence of recipient LCs and acute GVHD. Owing to the fact that acute GVHD is the most significant risk factor for chronic GVHD, high donor LC engraftment at 100 days is also more likely to be associated with chronic GVHD, although this has not been tested. Conversely, patients without acute GVHD are more likely to retain recipient LCs. Whether the level of recipient LCs remaining predicts the incidence of chronic GVHD is the question posed by Adani and colleagues in *Experimental Dermatology*. *Other reports suggest 90% engraftment may occur even in the absence of GVHD.

A recent paper in *Experimental Dermatology* examines a new aspect, the relationship between persistent recipient LCs and the occurrence of chronic GVHD (9). Perhaps mindful of the caveat just described, attention was focused on a defined subset of patients who had not experienced acute GVHD. In this way, a specific question could be asked whether more recipient LCs at day 100 predicted a greater risk of subsequent chronic GVHD. Twenty-two patients were free of GVHD at day 100, but the 6/22 patients who developed chronic GVHD had a similar level of recipient LC to those who did not (59%; range 22–95%).

The surprising finding is not the lack of an association with chronic GVHD, which is postulated to depend more on indirect antigen presentation by donor-derived DCs (10–12), but the high level of persistent recipient LCs reported. Although a lack of acute GVHD would favour retention of recipient LCs, previous reports had indicated that recipient LCs were largely eliminated by day 100 post-transplant (2,7,8). Andani and colleagues highlight a technical difference between their *in situ* analysis and studies showing high donor chimerism in LCs isolated from epidermal sheets by migration (7,8). Migration is an excellent way to isolate up to 1000 LCs from a small skin biopsy and to distinguish them from contaminating keratinocytes. A criticism of this approach is that *in vitro* culture might somehow favour the detection of donor-derived LCs. However, there is no *a priori* reason to justify this, and early after transplantation, it is possible to recover high numbers of recipient LCs by migration.

No technique is perfect, and *in situ* methods are liable to overestimate the proportion of recipient LC nuclei because the intricate branching of LC membrane can lead to the inadvertent inclusion of recipient keratinocyte nuclei. This problem may be minimized using the more intense peri-nuclear distribution of Langerin to detect LC nuclei; Langerin also has the distinct advantage of protease resistance that permits immunofluorescence to be performed after FISH. In addition, Z-stack reconstructions ensure that scored nuclei are completely encapsulated in LC membrane, and post-GVHD skin may be used to provide controls in which a very high proportion of donor LCs is expected. Using these approaches, high donor LC engraftment was also recently observed in a study incorporating a large cohort of minimally conditioned patients (13).

These considerations aside, Andani and colleagues have analysed a homogenous population of patients without GVHD, and their conclusion is that there is significant persistence of recipient LCs in the absence of skin inflammation. This is certainly in keeping with the reports of human limb transplantation, in which donor LCs survive long-term (14), and highlights the many clinical variables that must be accommodated when interpreting human studies. A critical factor is the definition of GVHD, because subclinical skin inflammation may promote donor LC engraftment (13). Previous reports would suggest that without GVHD, day 100 LC engraftment has a median of 90–95%, although the outliers of these studies overlap with the new data and are unlikely to be statistically separable (7,8,13).

The role of LCs in murine transplantation models is still debated. While it is clear that LCs may trigger acute GVHD if they are the only recipient APCs remaining (5,15), they may be deleted without consequence in other models (16). In a third scenario of mixed chimerism, LCs play a critical local role in epidermal inflammation (17). A recent trial of UV light treatment during human transplantation concluded that a lower risk of GVHD was associated with a reduction in LC numbers (18). Humans with *GATA2* mutation lacking DCs but retaining LCs and macrophages also still experience GVHD (19–21).

A new facet of GVHD research is the potential of stable recipient macrophages to promote GVHD. In human transplantation, macrophages outlive LCs and can stimulate allogeneic memory T-cell responses *in vitro* (22). In mice, it has now been formally demonstrated that tissue macrophages, like LCs, may also be maintained without bone marrow-derived precursors, leaving many models of GVHD pathogenesis open to reinterpretation (23–25).

In conclusion, we know that many recipient LCs are intact at the moment that donor T cells are infused into humans, and it remains difficult to rule out a contribution of recipient LC-mediated antigen presentation to acute GVHD. However, despite the persistence of many investigators, it remains increasingly hard to envisage that measurements of post-transplant LC chimerism will lead to useful clinical decisions.
